# Outbreak of *Chlamydia psittaci* Infection in a Commercial Psittacine Breeding Aviary in Argentina

**DOI:** 10.3390/ani14131959

**Published:** 2024-07-02

**Authors:** María Belén Riccio, Jorge Pablo García, María Laura Chiapparrone, Juliana Cantón, Claudio Cacciato, Javier Anibal Origlia, María Estela Cadario, Santiago Sain Diab, Francisco Alejandro Uzal

**Affiliations:** 1 Servicio de Diagnóstico Veterinario FCV Tandil, Facultad de Ciencias Veterinarias, Universidad Nacional del Centro de la Provincia de Buenos Aires, Tandil B7000GHG, Argentina; jorge@vet.unicen.edu.ar; 2 Laboratorio de Microbiología Clínica y Experimental, Centro de Investigación Veterinaria de Tandil (CIVETAN) (UNCPBA-CICPBA-CONICET), Facultad de Ciencias Veterinarias, Universidad Nacional del Centro de la Provincia de Buenos Aires, Tandil B7000GHG, Argentina; mlchiapp@vet.unicen.edu.ar (M.L.C.); jcanton@vet.unicen.edu.ar (J.C.); cacciato@vet.unicen.edu.ar (C.C.); 3 Cátedra de Patología de Aves y Pilíferos, Facultad de Ciencias Veterinarias, Universidad Nacional de La Plata, La Plata B1900BVB, Argentina; javieroriglia@yahoo.com; 4 INEI-ANLIS «Dr. Carlos G. Malbrán», Ciudad Autónoma de Buenos Aires, Buenos Aires B1282AFF, Argentina; mec135@yahoo.com.ar; 5 Department of Biomedical Sciences and Pathobiology, Virginia-Maryland College of Veterinary Medicine, Blacksburg, VA 24061, USA; santiagodiab@vt.edu; 6 California Animal Health and Food Safety Laboratory, School of Veterinary Medicine, University of California Davis, San Bernardino, CA 92408, USA; fauzal@ucdavis.edu

**Keywords:** outbreak, psittacine breeding aviary, Argentina, *Chlamydia psittaci*, chlamydiosis

## Abstract

**Simple Summary:**

Chlamydiosis, caused by the bacterium *Chlamydia psittaci*, commonly affects psittacine birds and can spread to humans. It is highly contagious among birds, manifesting in various form with clinical signs like respiratory distress, lethargy, and diarrhea. In humans, psittacosis presents with flu-like symptoms, including fever, headache, and respiratory difficulty; it can be severe or, if left untreated, fatal. The potential transmission of psittacosis from infected birds to humans, primarily through the inhalation of contaminated dust or aerosols, possesses a threat to human health. This article describes an outbreak of chlamydiosis in a commercial psittacine breeding aviary in Buenos Aires, Argentina, in 2021. The outbreak led to the deaths of different bird species, all showing weight loss, diarrhea, and respiratory distress. Necropsies revealed severe internal conditions, and histological examination confirmed the infection. The diagnosis was verified through immunohistochemistry and molecular techniques, identifying genotypes A and B of *Chlamydia psitacci*.

**Abstract:**

Chlamydiosis, caused by *Chlamydia psittaci* is a bacterial infection found in at least 465 species of birds worldwide. It is highly contagious among birds and can spread to humans. In birds, the disease can manifest itself in acute, subacute, and chronic forms with signs including anorexia, diarrhea, lethargy, weight loss, or, occasionally, mucopurulent or serous oculonasal discharge. This article describes an outbreak of chlamydiosis that occurred in a commercial psittacine breeding aviary in 2021 in Buenos Aires province, Argentina. In total, 16 juvenile blue-fronted parrots, more than 60 blue-fronted parrot chicks, and 2 adult macaws died during the outbreak. In all cases, clinical signs were weight loss, diarrhea, yellowish green excrement, and respiratory distress. The necropsy of four juvenile blue-fronted parrots, two blue-fronted parrot chicks, and two adult macaws revealed cachexia, hepatomegaly, splenomegaly, splenic petechial hemorrhages, ascites, pulmonary edema, and hydropericardium. Histologically, multifocal lymphoplasmacytic and heterophilic airsaculitis, multifocal lymphoplasmacytic and necrotizing hepatitis with intracytoplasmic elementary bodies, multifocal necro-heterophilic hepatitis, multifocal lymphoplasmacytic nephritis, and diffuse heterophilic pneumonia were found. A presumptive diagnosis was established based on gross and microscopic lesions, and it was confirmed using immunohistochemistry and polymerase chain reactions. The sequencing and phylogenetic analysis of the *ompA* gene revealed genotype A and B of *Chlamydia psittaci*.

## 1. Introduction

Chlamydial infections caused by widespread obligate intracellular Gram-negative bacteria from the *Chlamydiaceae* family are associated with a wide spectrum of clinical symptoms in livestock, companion animals, wildlife, and various exotic species including several birds species [1,2,3]. In addition, some chlamydial species, such as *Chlamydia abortus* and *Chlamydia psittaci*, are capable of causing zoonotic infections [4,5]. 

In humans, *Chlamydia psittaci* (*C. psitacci*) causes a severe disease, known as psittacosis, which, in most cases, can lead to pneumonia and significant mortality if left untreated [6,7,8,9]. Psittacosisis is a significant threat to public health and is considered an occupational zoonosis of pet shop owners, zookeepers, veterinarians, abattoir workers, bird breeders, and pet bird enthusiasts [2,10,11].

At least 465 bird species belonging to 30 orders can have chlamydiosis. However, psittacidae (lories, parakeets, parrots, and cockatoos) and columbiformes (pigeons) are the most frequently affected. Primary reservoirs for zoonotic psittacosis are psittacine pets, as well as wild and racing pigeons [12].

*C. psittaci* replicates using a biphasic developmental cycle within cytoplasmic inclusion vacuoles of eukaryotic cells. This cycle involves two morphologically different forms, as follows: the infectious extracellular form, termed the elementary body (EB), and the reproductive reticulated body (RB). The EB enters a host cell through receptor-mediated endocytosis. This process, which lasts several hours, results in the conversion of the EB into a reticulate body (RB). The RB divides via binary fission to produce more EBs. This cycle continues for several hours after infection, at which time the host cell lyses, releasing several hundred EBs, RBs, and intermediates [13].

Chlamydiosis causes a range of clinical signs in birds, including respiratory difficulty, diarrhea, and lethargy, and it can also lead to chronic or asymptomatic infections. Some infected birds may appear healthy, but shed the organism intermittently. Shedding may be exacerbated by internal factors such as the bird’s immune status, in addition to external factors such as stress, reproductive activities, relocation, shipping, crowding, and cold temperature. The severity of clinical signs varies significantly, influenced by the species and age of the birds, as well as the specific strains of the pathogen involved [2].

In psittacine birds, gross lesions include splenomegaly, hepatomegaly, enteritis, sinusitis, air sacculitis, pericarditis, and conjunctivitis. Microscopic lesions of chlamydiosis in birds include necrotizing changes in several organs associated with chlamydial intracytoplasmic elementary bodies and the infiltration of heterophils, macrophages, and plasma cells [14,15,16].

*Chlamydia psittaci* infection in birds has been reported previously in many countries [1,17,18,19,20]. In recent years, there has been a global increase in the number of reported cases of avian chlamydiosis in both birds and humans [9,21,22,23].

The analysis of *C. psittaci ompA* gene sequences is crucial for genotype determination [24]. In the studied psittacidae population, the circulating genotypes are identified as A and B. Initially, *C. psittaci* was categorized into nine genotypes—A-F, E/B, M56, and WC, with the later proposal of eight new genotypes [1,24]. Genotype A is prevalent among psittacine birds, but is also found in turkeys, ducks, pigeons, and Passeriformes. Genotype B is endemic in pigeons but can infect various bird species including chickens, turkeys, ducks, Psittacidae, and Passeriformes. Waterfowl are commonly infected with genotype C and E/B strains, while turkeys can harbor genotypes D and C. Genotype E is found in ducks, pigeons, ostriches, and rheas, and genotype F is in psittacine and turkey isolates [1,25,26,27].

This article describes a large outbreak of psittacosis in a commercial breeding aviary in Buenos Aires, Argentina, and emphasizes the need to monitor and quickly identify the disease to minimize its impact in birds and prevent the spread to people at risk.

## 2. Materials and Methods

### 2.1. Case Presentation

The outbreak occurred between January and April 2021 at a new facility of a commercial psittacine breeding aviary in Buenos Aires province, Argentina. This aviary specializes in the conservation, breeding, and export of blue-fronted Amazon parrots. It also acts as a rescue center, receiving and rehabilitating parrots and macaws that have been confiscated from the illegal trade. The population of psittacine birds included blue-fronted parrots (*Amazona aestiva*), red-and-green macaws (*Ara chloropterus*), and scarlet macaws (*Ara macao*). Previously, they were in other facilities with outdoor cages. The new aviary has a ground floor with the breeding and quarantine area, and the first floor houses the juvenile and adult birds in indoor cages. These cages are 7.5 m long, 1.5 m wide, and 2.4 m high. The parrots have ad libitum access to fruit, vegetable, and seeds. Water is provided *ad libitum* in bowls. Excess food is removed daily, the floors are dry cleaned, the trays are washed, and fresh water is replenished.

During January and April 2021, 63 out of 120 few-days-old blue-fronted parrot chicks, 16 out of 100 juvenile blue-fronted parrots, 1 1.5-year-old red-and-green macaw (*Ara chloropterus*), and a similarly aged juvenile scarlet macaw (*Ara macao*) died.

The affected birds exhibited lethargy, refusal to eat, regurgitation, depression, cachexia, anorexia, respiratory distress, diarrhea, and yellowish green excrements. Despite treatment, most birds with these symptoms died quickly.

The mortality rate of the outbreak was 52.5% in blue-fronted parrot chicks, 16% in juvenile blue-fronted parrots, and 25% in green-and-red macaws. The only scarlet macaw specimen also died.

After confirming the diagnosis of chlamydiosis in mid-April, a new therapeutic approach consisting of the oral administration of oxytetracycline at a rate of 300 mg/kg in food, per animal, every 24 h for 45 days was started.

To evaluate the effectiveness of this treatment, a triple mucosal swab from conjunctiva, choanae, and cloaca obtained from two adult blue-fronted parrots previously tested positive for *C. psitacci* were collected and submitted for *Chlamydia* spp. real-time PCR analysis.

### 2.2. Necropsy and Ancillary Tests

Necropsies were performed on four juvenile blue-fronted parrots (P1, P2, P3, P4), two blue-fronted parrot chicks (C1, C2), one red-and-green macaw, and one scarlet macaw. All birds were weighed. Sterile organ samples, including lung, liver, bursa of Fabricius, air sac, and laryngeal swabs, were analyzed for *Trichomonas* and *Salmonella* sp. from P1 and C1. Additionally, *Salmonella* cultures were performed on liver samples from all birds, except for macaws. Lung and liver samples from all birds, excluding macaws, were submitted for fungal culture. Selected organs, such as liver, lung, air sacs, spleen, intestine, and kidneys, were processed for histopathology. Triple mucosal swabs from the conjunctiva and choanae, or pooled organ samples from liver, lung, and spleen, were submitted for real-time PCR to detect *C. psittaci*, Psittacid Herpesvirus, and Avian Polyomavirus. Paraffin-embedded spleen and kidney samples from P2, P3, and a scarlet macaw were submitted for immunohistochemistry (IHC).

### 2.3. Microbiology

For the detection of aerobic and microaerophilic mesophile bacteria, the organ and swab samples submitted from juvenile and chick blue-fronted parrots were cultured in two culture media—tryptone soy agar supplemented with 10% bovine blood, under aerobic and microaerophilic (5% O_2_, 10% CO_2_, and 85% N_2_) conditions for 24 and 48 h, and MacConkey agar at 37 °C, under aerobic conditions, for 24 h. The samples were cultured in Selenite broth at 37 °C and 42 °C as a pre-enrichment medium and were sub-cultured at 24 h and 48 h in *Salmonella–Shigella* agar, and were incubated for 24 h at 37 °C, all of this in aerobic conditions. The isolations were identified through colonies and bacterial morphologies, Gram-stain, and biochemical tests [13,28].

For *Trichomonas* sp., the samples were cultured in Diamond (TYM) medium at 37 °C in aerobic conditions and were observed every 24 h for 7 days. For fungi, the samples were cultured in Sabouraud agar at 25 °C in aerobic conditions and were observed every 24 h for 10 days.

The antibiotic susceptibility test was performed using the disc diffusion method (Kirby Bauer) according to Clinical and Laboratory Standards Institute guidelines (CLSI, 2020).

### 2.4. Histopathology

The organs submitted (liver, spleen, air sacs, heart, intestine, and kidneys) from all birds were fixed in a 10% neutral buffered formalin solution. Tissues were embedded in paraffin, sectioned at 4 μm, mounted on glass slides, and stained with hematoxylin and eosin (H&E) using the routine method. Selected kidney sections from the red-and-green macaw were stained with the Gimenez method.

### 2.5. Inmunohistochemistry

Selected sections of liver and spleen from the scarlet macaw, and the spleen, liver, and kidney from parrots P2 and P4 were processed using immunohistochemistry (IHC) following standard operating procedures at the California Animal Health and Food Safety Laboratory, University of California, Davis, USA. Briefly, paraffin-embedded tissues were sectioned at 4 mm and were mounted on charged slides, deparaffinized, and hydrated, quenching endogenous peroxidase with 3% hydrogen peroxide. Slides were then placed in 0.4% acidulated pepsin for antigen retrieval for 15 min in a 37 °C water bath and were subsequently rinsed in running lab-grade water for 3–5 min and then buffer solution (TBS-Tween, Saint Louis, MI, USA) for 3–10 min. The primary antibody (ViroStat #1681 (Le Pontet, Vaucluse, France), *Chlamydia pecorum*-LPS (genus specific, cross-reactive with *Chlamydia pneumonia* and *Chlamydia psittaci*), and non-specific reagent were applied for 30–60 min. Sections were rinsed in buffer solution, and a secondary antibody (Dako Envision + K4001, Santa Clara, CA, USA) was applied for 30 min. After rinsing in buffer solution again, a chromogen (AEC, ready-to-use, Dako K3464) was applied to the sections for 10–20 min. Slides were then rinsed in lab-grade water and were subsequently placed in Mayer’s hematoxylin for 1–4 min. After rinsing with lab-grade water again, slides were blued in Scott’s tap water for 1–2 min. Slides were finally rinsed in running lab-grade water and coverslips with aqueous mounting media were applied. All incubations were at room temperature.

### 2.6. Chlamydia Isolation Procedure

The isolation of *Chlamydia* in VERO cell cultures was attempted for some of the samples stored in transport medium, following previously described procedures under biosafety level 3 precautions [29].

### 2.7. Polymerase Chain Reaction

Fresh and frozen tissues (liver, spleen, and lung) and trimucosal swabs were subjected to DNA extraction. Approximately 25 µg of the homogenated pool of organs from the necropsied birds were used for DNA extraction. Each swab was resuspended in 600 µL of phosphate-buffered saline (PBS) and were homogenized using a vortex; DNA extraction was carried out from 200 µL of each sample. For both types of samples, the High Pure PCR Template Preparation Kit (Roche, Mannheim, Germany) was used according to the manufacturer’s instructions. Extracted DNA was stored at −20 °C until use.

For the detection of *C. psittaci*, real-time PCR was performed for the *ompA* gene target, as described by Pantchev et al. 2009 [30]. Those samples with a CT bellow 38 were considered positive. Specific real-time PCR was also performed for the detection of Psittacid Herpesvirus and Avian Polyomavirus [31]. Endpoint PCR was performed on the samples that were positive for *C. psittaci* real-time PCR for the partial amplification of the *ompA* gene (approximately 1050 bp) [24]. Positive and negative control samples were included in each PCR reaction. The endpoint PCR products were analyzed using electrophoresis in 2% agarose gels stained with Syber Safe DNA gel Stain (Invitrogen, Waltham, MA, USA) and a 100 bp DNA Ladder (Genbiotech, Buenos Aires, Argentina) as a marker, and they were visualized under ultraviolet light. PCR products were purified with DNA Clean & Concentrator (Zymo Research, Tustin, CA, USA) and were sent for sequencing using Sanger technology.

For the alignment and obtaining of the consensus sequences, the SeqAssem program was used. Consensus sequences were compared using BLASTn 2.2.19 with other fragments of the *Chlamydia* spp. *ompA* gene obtained from GenBank. The dendrogram was constructed using the Tree Explorer module of the MEGA7 program, employing the Neighbor-joining method with the p-distance parameter. Statistical support was determined through nonparametric bootstrapping with 1000 pseudo-replications [32].

The scheme developed by Pannekoek et al. [33] targeting seven housekeeping genes (*gatA, oppA, hflX, gidA, enoA, hemN*, and *fumC*) was used for Multi-Locus Sequence Typing (MLST) genotyping. Genes were amplified and sequenced using primers for the *C. psittaci* scheme available on the *Chlamydiales* PUBMLST database (http://pubmist.org/chlamidiales, accessed on 26 April 2024) and following the protocols described by Jelocnik et al. [34] and Mattman et al. [25]. The PCR products’ visualization, purification, and sequencing were performed as describe above for endpoint PCR. The SeqAssem program was used for the alignment and obtaining of the consensus sequences. The allele designation for each aligned sequence and the allelic profile to determine sequence type (ST) was obtained using the “sequence query” and “Search by combination of allele” options, respectively, from the *Chlamydiales* PubMLST database (http://pubmist.org/chlamidiales, accessed on 26 April 2024) [34]. The MLST sequences generated were deposited in the previously mentioned database.

## 3. Results

### 3.1. Necropsy

**Figure 1:**
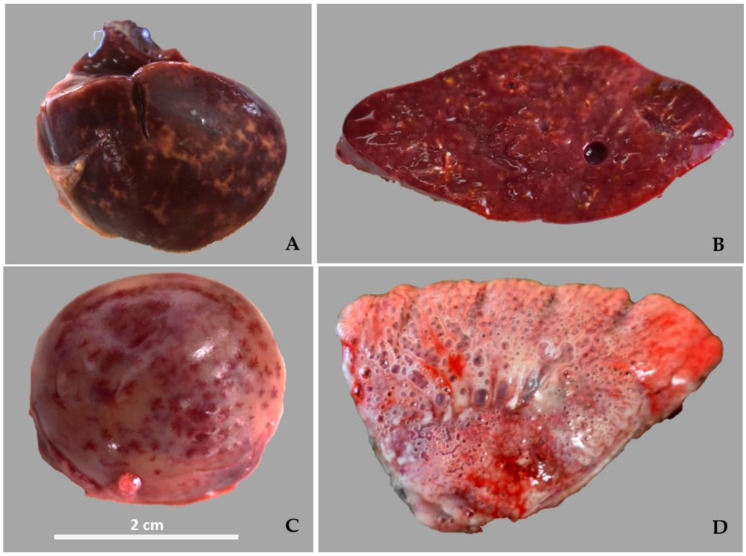
Post-mortem lesions of organs collected from birds affected by *Chlamydia psittaci* in a commercial psittacine breeding aviary in Argentina. (**A**) Marbled surface of the liver with white, irregular, yellowish flat spots of variable size. Juvenile blue-fronted parrot. (**B**) Marbled cross section of the liver from a red-and-green macaw. (**C**) Enlarged, pale, and mottled spleen from scarlet macaw. (**D**) The lung is edematous and moderately congested.

Grossly, all birds were in poor nutritional condition, with no fat reserves, prominent keels, and atrophy of the pectoral muscles. The liver of all birds, with the exception of chick blue-fronted parrots, was enlarged and the surface cross section had multifocal, irregular, variably sized, tan-to-yellow, non-raised areas of discoloration. In both macaws, the spleen was enlarged, pale, and mottled by multiple dark red foci. The pericardium of P 3 was opaque and the pericardial sac was filled with a moderate amount of yellow liquid (0.5–1 mL). The coelomic cavity of P2 was filled with 2 to 3 mL of a cloudy yellow liquid. In addition, congested and edematous lungs were observed in all birds (Figure 1).

### 3.2. Microbiology

Cultures for *Trichomonas* sp. and fungi were negative in all birds tested.

*Pseudomonas aeruginosa* was isolated and identified from all the samples of P1, lung of P2, and lung of C1. *Salmonella* sp. was isolated from the liver of both blue-fronted parrot chicks, as well as from the laryngeal swab of C2. In addition to *Pseudomonas* and *Salmonella* isolated from the samples of both nestlings, *Escherichia coli*, *Klebsiella* sp., and *Proteus* sp. were also isolated.

### 3.3. Histology

**Figure 2:**
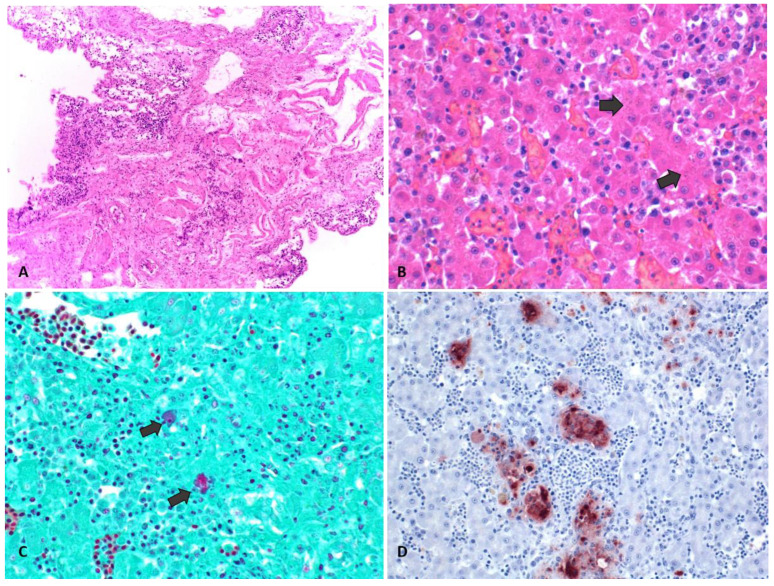
Microscopic lesions of organs collected from birds affected by *Chlamydia psittaci* in a commercial psittacine breeding aviary in Argentina (**A**) Air sac: Lymphoplasmacytic and necrotizing multifocal airsacculitis 20X H&E. (**B**) Liver: Intracytoplasmic elementary bodies in the cytoplasm of hepatocytes and mononuclear cells (black arrows) 40X H&E. (**C**) Liver: elementary bodies in the cytoplasm of hepatocytes, Gimenez stain (Black arrows). (**D**) Positive Chlamydia spp. IHC on liver.

The main microscopic findings are summarized in Table 1. Hepatitis, splenitis, air sacculitis, pulmonary congestion, and edema were the main findings in all animals examined (Figure 2). In addition, nephritis and pneumonia were found in C1 and green-and-red macaws.

Figure 2 shows the microscopic and immunohistochemical findings.

### 3.4. Immunohistochemistry

IHC for *Chlamydia pecorum* cross-reacting with *Chlamydia* spp. was positive on the liver, kidney, and spleen sections of two juvenile blue-fronted parrots (P2 and P4) and the scarlet macaw tested.

### 3.5. Chlamydia Isolation

Isolation was obtained only from samples from the scarlet macaw that had been adequately preserved prior to processing. Identification of the strain was carried out with the specific real-time PCR of *C. psittaci*.

### 3.6. Polymerase Chain Reaction

From swab samples of cloaca, choanae, and conjunctiva, as well as of the pooled organ samples (liver, spleen, and lung) of juvenile blue-fronted parrots (P2, P3, and P4) and blue-fronted parrot chicks (C1 and C2), bacterial nucleic acid belonging to *C. psittaci* DNA was detected. In conjunctiva, choanae, and cloacal swab samples, as well as liver, spleen, and lung pool samples of both macaws, bacterial nucleic acid belonging to *C. psittaci* was detected. No positive results were shown for Psittacine Herpesvirus or Avian Polyomavirus in the tested samples.

**Figure 3:**
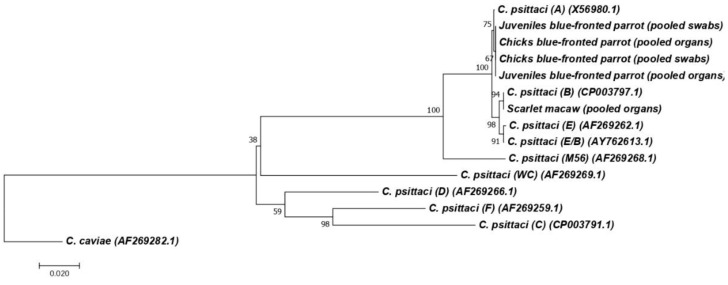
Neighbor-joining dendrogram based on comparison of the *ompA* gene fragment (about 1030 bp) of *Chlamydia psittaci*. Representative sequences from the different *C. psittaci* genotypes were included. Samples belonging to this study were pooled organs from juvenile blue-fronted parrots (P2, P3, and P4), pooled trimucosal swabs from blue-fronted parrot chicks (C1 and C2), pooled trimucosal swabs from juvenile blue-fronted parrots (P2 and P3), pooled organs from blue-fronted parrot chicks (C1 and C2), and pooled organs from a scarlet macaw. The GenBank accession numbers were PP751030, PP751031, PP751032, PP751033, and PP706175, respectively. The evolutionary distances were computed using the Kimura 2-parameter method and are in the units of the number of base substitutions per site. Numbers above branches are Bootstrap values as a percentage of 1000 pseudo replicates. *C. caviae* GPIC was used as an outgroup. The scale bar shows the sequence diversity percentage [32].

The partial *ompA* gene could be amplified and sequenced directly from the clinical specimens obtained from the swab samples of cloaca, choanae, and conjunctiva of juvenile blue-fronted parrots (P2 and P3), blue-fronted parrot chicks (C1 and C2), pooled organ samples (liver, spleen, and lung) from juvenile blue-fronted parrots (P2–P4), blue-fronted parrot chicks (C1 and C2), and of the isolate obtained from the scarlet macaw. All sequences obtained from the blue-fronted parrots were identical to each other and were grouped together with the genotype A reference strains. Moreover, the sequence obtained from the scarlet macaw (Genbank accession number PP706175) was similar to the reference genotype B sequence (Figure 3).

The MLST analysis was carried out only on the isolated strain from the scarlet macaw and this showed that it corresponded to ST27.

The mucosal swab samples obtained after the treatment of two juvenile blue-fronted parrots gave a negative result.

## 4. Discussion

*Chlamydia psittaci* is an important zoonotic pathogen that could infect a wide range of hosts, although the most common hosts of *C. psittaci* are birds, especially parrots. Our results are consistent with findings reported by other authors [12,35,36].

After reviewing the literature available from PubMed, Google Scholar, and Web of Science, we could not find any reports of an outbreak of avian chlamydiosis in a commercial psittacine breeding aviary in Argentina. However, many publications indicate the detection of *C. psittaci* in various provinces of Argentina, in both bird and human samples [37,38,39,40].

*C. psittaci* transmission occurs primarily by close contact when the agent is shed in nasal discharges and feces. fecal shedding is intermittent and it is exacerbated when animals are stressed. During natural infection, virulence of the strain, infection dose, and host immune status greatly affect bacterial excretion [41].

In all of the *Chlamydia*-positive birds included in this study, clinical signs compatible with chlamydiosis [41,42] were observed and submitted to necropsy. All birds analyzed were emaciated which, along with the presence of hepatic and splenic lesions, is consistent with chlamydiosis, as previously reported [2,42,43]. For the differential diagnosis, psittacid Herpesvirus 1 (Pacheco´s parrot disease) and Avian Polyomavirus infection should be considered. In this study, PCR for both diseases was negative in all birds analyzed. Regarding the isolation of *Salmonella* and *Pseudomona aeruginosa*, we believe that the co-infection may have contributed to increased mortality. However, only 37% of the birds analyzed had a co-infection, which suggests that *Chlamydia* infection can produce a high mortality rate on its own.

The presumptive diagnosis of chlamydiosis is based on gross and microscopic lesions and confirmed using PCR [44,45] and/or immunohistochemistry. The isolation of *C. psittaci* from a clinical specimen is a very good confirmatory test but is not frequently performed by veterinary diagnostic laboratories because of its technical difficulty [46]. In addition, culturing is hampered by limited sensitivity, previous antibiotic use, and strict biosafety regulations [47]. We were able to perform the isolation from a pooled sample of organs (liver, spleen, and lung) of a scarlet macaw that was refrigerated in SPG medium. Unfortunately, the rest of the samples were frozen at −20 °C without preservation medium prior to being sent to the laboratory, so their culture was not performed. From the isolation, it was possible to obtain DNA in good concentration and quality, which allowed the MLST characterization of the strain.

Using the genotyping strategy based on the ompA gene, we were able to determine that two genotypes were involved in the outbreak studied by us. We were able to determine that the samples from blue-fronted parrots had genotype A. This genotype is often involved in outbreaks of avian chlamydiosis with high mortality in psittacines [2], as was in our case. On the other hand, we found that genotype B in the scarlet macaw was associated with severe clinical signs and lesions. Previously, this genotype was found in an African gray parrot (*Psittacus erithacus erithacus*) kept as a pet that presented avian chlamydiosis [27]. Furthermore, in Argentina, the circulation of these two genotypes has been confirmed in previous studies [38,48].

Strains of *C. psittaci* had not been previously characterized using MLST in Argentina; in our study, we were able to determine that the strain isolated from the scarlet macaw corresponded to ST27. This ST has been found in pigeons, passerines, parrots, penguins, and horses in North America, Europe, Australia, and New Zealand. In Australia, it has been associated with abortions in horses, which provides evidence of potential host-switching and spillover between different hosts of this ST [24,25,49].

The epidemiological characterization of animal and human samples provides information on prevalent genotypes in human infections, the likely avian sources, and aids in surveillance and outbreak management [47]. Psittaciformes are a major source of the *Chlamydia* responsible for psittacosis in humans [36]. Human psittacosis ranges from mild to severe systemic illness, with genotype A considered to be the most virulent and closely associated with human cases and psittacine birds [48,50]. However, genotype B, mainly associated with pigeons, also poses zoonotic transmission risks [36,47]. There is little information in Argentina demonstrating the genotypes involved in cases of zoonotic transmission. Cadario et al. [48] found genotype A in two cases of human psittacosis and managed to determine the same genotype in the psittacidae considered as the source of infection.

During the outbreak, a worker displayed symptoms suggestive of psittacosis, but amidst the COVID-19 pandemic, was diagnosed with SARS-CoV-2 based solely on clinical presentation without confirmatory tests. The clinical features of SARS-CoV-2 and *C. psittaci* infection can overlap, posing challenges for accurate differential diagnosis [51,52]. Co-infections of SARS-CoV-2 and *Chlamydia* spp. have also been documented [51,52]. The worker was eventually treated with doxycycline after an X-ray showed a lung lesion compatible with bacterial pneumonia, but unfortunately no samples were obtained to make a confirmatory diagnosis.

Although the exact point of entry of *Chlamydia* into the aviary remains unknown, we speculate that the source of infection was the introduction of a different sick bird, which could explain the independent introduction of the pathogens, as the aviary also serves as a rescue center for confiscated birds. The proximity of the breeding and quarantine areas may have increased the risk of transmission. Additionally, we speculate that the detection of both genotypes, genotype A and B, may be due to the birds contracting the infection when the aviary was located at its previous facilities, where the cages were kept outdoors. The area where the aviary is located is a rural and rugged area with an abundant presence of wildlife. Among the wild birds, the monk parakeet (*Myiopsitta monachus*), the burrowing parrot (*Cyanoliseus patagonus*) and various species of pigeons (*Columba livia*, *Zenaida auriculata*, *Patagioenas picazuro*), in addition to a wide variety of passerines, are very abundant in the place, and free-living wild birds can act as reservoirs and shedders of *C. psittaci* and other chlamydial agents [17,24,25]. It is well known that the main sources of *C. psittaci* genotype A are psittacidae, and that of genotype B are pigeons. Furthermore, passerines are also recognized as possible hosts for both genotypes [2].

Regarding treatment, oxytetracycline was administered orally through food. Although we are aware of the disadvantages of this route of administration, it was chosen to reduce the stress associated with individual parenteral treatments. The effectiveness of using oxytetracycline as the antibiotic of choice during the outbreak has been previously demonstrated by other authors [53,54]. Administering drugs through food or drinking water reduces the stress associated with individual handling and injections, which can be particularly challenging and distressing for birds, but it is important to remember that acutely ill birds that stop eating and drinking are less likely to recover.

## 5. Conclusions

The present study is the first report of an outbreak of chlamydiosis in a commercial psittacine breeding aviary from Buenos Aires, Argentina. The pathogen, predominantly affecting birds, particularly parrots, demonstrates zoonotic potential. Zoonotic transmission, particularly involving genotypes A and B, emphasizes the importance of surveillance and diagnostic efforts. Understanding the prevalent genotypes in regions like Argentina is crucial for effective public health interventions and outbreak management. In conclusion, this analysis highlights the complex epidemiology of *C. psittaci* infections, emphasizing the critical role of surveillance, genotype characterization, and differential diagnosis in mitigating the disease’s impact on both avian and human health.

## Figures and Tables

**Table 1:** Histology results for samples collected from parrots, chicks, and macaws.

Lesion Observed	P1	P2	P3	P4	C 1	C 2	Green-and-Red Macaw	Scarlet Macaw
Multifocal lymphoplasmacytic, heterophilic, and necrotizing splenitis with intracytoplasmic elementary bodies								
	Yes	Yes	Yes	Yes	Yes	Yes	Yes	Yes
							
Pulmonary congestion with edema	Yes	Yes	Yes	Yes	Yes	Yes	Yes	Yes
Multifocal, lymphoplasmacytic, and necrotizing hepatitis with intracytoplasmic elementary bodies								
	No	Yes	No	No	Yes	No	No	Yes
							
							
Multifocal necro-heterophilic hepatitis	No	No	No	Yes	No	Yes	No	No
Multifocal lymphoplasmacytic, heterophilic airsacculitis	Yes	Yes	No	No	No	Yes	Yes	Yes
Multifocal lymphoplasmacytic nephritis	No	No	No	No	No	No	Yes	No
Heterophilic pneumonia	No	No	No	No	Yes	No	No	No

## Data Availability

The sequences generated in this study are available in GenBank under Accession Numbers PP751030, PP751031, PP751032, PP751033, and PP706175. The MLST sequences generated in this study are deposited under the ID 5164 in PubMLST/Chlamydiales (https://pubmlst.org/chlamydiales/, accessed on 10 May 2024 and 26 April 2024 respectively).
